# Responses of Winter Wheat Yield and Water Use Efficiency to Irrigation Frequency and Planting Pattern

**DOI:** 10.1371/journal.pone.0154673

**Published:** 2016-05-12

**Authors:** Chengyue Bian, Changjian Ma, Xinhui Liu, Chao Gao, Quanru Liu, Zhenxing Yan, Yujie Ren, Quanqi Li

**Affiliations:** 1 College of Water Conservancy and Civil Engineering, Shandong Agricultural University, Tai’an 271018, China; 2 Department of Water Conservancy Engineering, Shandong Water Conservancy Vocational College, Rizhao 276800, China; University of Vigo, SPAIN

## Abstract

A suitable planting pattern and irrigation strategy are essential for optimizing winter wheat yield and water use efficiency (WUE). The study aimed to evaluate the impact of planting pattern and irrigation frequency on grain yield and WUE of winter wheat. During the 2013–2014 and 2014–2015 winter wheat growing seasons in the North China Plain, the effects of planting patterns and irrigation frequencies were determined on tiller number, grain yield, and WUE. The two planting patterns tested were wide-precision and conventional-cultivation. Each planting pattern had three irrigation regimes: irrigation (120 mm) at the jointing stage; irrigation (60 mm) at both the jointing and heading stages; and irrigation (40 mm) at the jointing, heading, and milking stages. In our study, tiller number was significantly higher in the wide-precision planting pattern than in the conventional-cultivation planting pattern. Additionally, the highest grain yields and WUE were observed when irrigation was applied at the jointing stage (120 mm) or at the jointing and heading stages (60 mm each) in the wide-precision planting pattern. These results could be attributed to higher tiller numbers as well as reduced water consumption due to reduced irrigation frequency. In both growing seasons, applying 60 mm of water at jointing and heading stages resulted in the highest grain yield among the treatments. Based on our results, for winter wheat production in semi-humid regions, we recommend a wide-precision planting pattern with irrigation (60 mm) at both the jointing and heading stages.

## Introduction

Wheat (*Triticum aestivum* L.) is the second largest crop in the world. Due to current water shortage issues, it is essential that the water use efficiency (WUE) of winter wheat be improved, while maintaining, or potentially increasing, grain yields. For example, the North China Plain occupies 39% of the country’s cultivated area; but only has 8% of the nation’s water resources, and this water shortage has seriously restricted the development of winter wheat production.

Determining a suitable irrigation frequency is an important step in being able to optimize winter wheat yield and WUE. Irrigation frequency can affect plant growth in various ways. Decreased irrigation frequency is an important technique used to improve WUE of paprika [1] and citrus [2]. In winter wheat, increased irrigation frequency results in low evapotranspiration [3]. Han et al. [4] revealed that by irrigating twice in the winter wheat growing season, grain yield could be increased; however, irrigation timing at the end of the growing season could decrease grain yield. Similarly, Li et al. [5] revealed that frequent irrigation late in the winter wheat growing season decreased WUE, and this was mainly due to changes in the vertical distribution of root density. Previous studies also suggested that, for winter wheat, a one-time irrigation of 120mm could produce a reasonable grain yield and WUE, and irrigation (60 mm) at both the jointing and heading stages significantly improved WUE [5, 6, 7].

Planting pattern also plays an important role in improving grain yield and WUE. Research in North India revealed that planting pattern could significantly increase both winter wheat and summer maize (*Zea mays* L.) grain yield and WUE [8]. In a North China study, furrow planting significantly increased winter wheat grain yield and WUE under water deficit conditions [5]. In addition, partial root zone irrigation was found to affect the growth and WUE of crops, such as summer maize [9], potato [10], and grape [11].

In recent years, a new planting pattern known as “wide-precision” has been broadly adopted. The new planting pattern has a sowing width of 6–8 cm (conventional sowing width is 3–5 cm), and, compared to conventional planting, a wide seed dispersal, as single grains are separated from each other instead of being planted in a line. In 2010, this new planting pattern produced the highest winter wheat grain yield in North China [12]. In the late stages of winter wheat growth, leaf area index was higher in the wide-precision plantings than in conventional-cultivation plantings [12]. Irrespective of irrigation schemes, both tiller number and WUE were significantly higher in the wide-precision plantings than in the conventional-cultivation plantings [6].

Under field conditions, 25–30% of available water is not used by the crop and lost as soil evaporation [13, 14]; therefore, reducing soil evaporation is crucial to increasing WUE. Applying the same irrigation volume, but reducing irrigation frequency, could effectively suppress soil evaporation; however, winter wheat yield may be reduced because of water limitations late in the growing season [5]. Under water deficit conditions, a wide-precision planting pattern, compared to a conventional-cultivation planting pattern, resulted in a significantly higher winter wheat grain yield and WUE, and this was mainly due to a notable increase in spike number [6]. Calculating tiller number is important for determining spike number, which is the yield component most closely correlated with winter wheat grain yield [15].These studies, however, have not reported the integrated effects of the planting patterns when combined with irrigation frequency on grain yield and WUE of winter wheat. Based on the aforementioned research, we hypothesized that altering the planting pattern and irrigation frequency may improve the WUE of winter wheat.

In the present study, we investigated the effect of planting pattern and irrigation frequency on winter wheat: (i) tiller numbers; (ii) soil water consumption and total water consumption; and (iii) grain yield and yield components. The results of this investigation could provide the theoretical basis and technical knowledge for the implementation of water efficient, high-yielding winter wheat planting pattern in semi-humid regions.

## Materials and Methods

### Experimental site

The experiment was conducted during the 2013–2014 and 2014–2015 winter wheat growing seasons at the Agronomy Station of Shandong Agricultural University (36°10′19″N, 117°9′03″E) in the North China Plain. In this semi-humid region, the annual precipitation is 697 mm, of which approximately 35% occurring during the winter wheat growing season. Each experimental plot was 9.0 m^2^, with a loamy soil and concrete slabs placed around each plot to prevent the lateral flow of soil water. Field capacity was 36%, wilting point was 10%, saturated water content was 43%, and the concentration of organic matter was 1%. Nutrient levels, measured in the 0–20 cm soil layer, were 65, 15, and 82 mg·kg^-1^ for N, P, and K, respectively. At the time of sowing, 38 g·m^−2^ of urea, 26 g·m^−2^ of diammonium hydrogen phosphate, and 21 g·m^−2^ of potassium sulfate were applied to the soil as basal dressing. A further 38 g·m^−2^ of urea was added at the jointing stage. On October 9, 2013, and October 7, 2014, winter wheat was hand-planted at a density of 222 plants·m^-2^, and was harvested on June 1, 2014, and June 9, 2015, respectively. The cultivar used for the experiment was “Jimai 22”, which is a commonly used cultivar in the North China Plain. The field study was performed with permission from the Agronomy Station of Shandong Agricultural University.

### Experimental design

**Fig 1:**
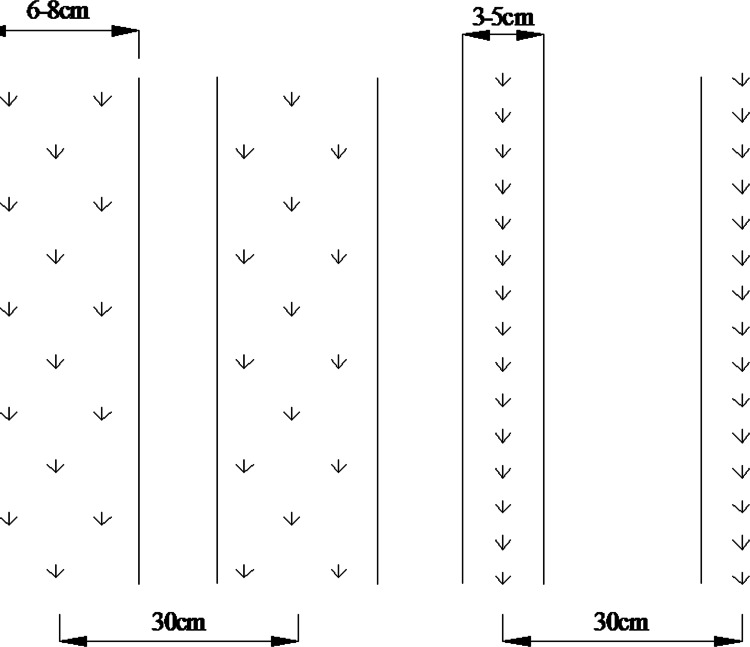
Schematic diagram of wide-precision planting and conventional-cultivation planting in this study.

A split-plot design was applied with two planting patterns in the main plots: wide-precision planting pattern (W) and conventional-cultivation planting pattern (C). Row spacing in each planting pattern was 30 cm apart, and sowing widths in the wide-precision and conventional-cultivation plantings were 6–8 cm and 3–5 cm, respectively. A schematic diagram of both planting patterns was provided in Fig 1. In the sub-plots, three different irrigation regimes were set-up: irrigation (120 mm) at the jointing stage (I1); irrigation (60 mm) at the jointing and heading stages (I2); and irrigation (40 mm) at the jointing, heading, and milking stages (I3). Jointing, heading, and milking stages were on March 30, April 15, and May 1, 2014 during the first growing season, and on March 28, April 24, and May 14, 2015 during the second growing season. The quantity of irrigation water applied during the growing seasons is presented in Table 1. Water was supplied to the plots through plastic pipes connected to a pump outlet, and a flow meter was used to measure the amount of irrigation water. The experiments were conducted in triplicate in a randomized block design, yielding a total of 18 sub-plots.

**Table 1:** Amount of irrigation water (mm) applied in the 2013–2014 and 2014–2015 winter wheat growing seasons.

Treatments	Jointing stage	Heading stage	Milking stage	Total irrigation
WI1	120	0	0	120
WI2	60	60	0	120
WI3	40	40	40	120
CI1	120	0	0	120
CI2	60	60	0	120
CI3	40	40	40	120

Note: W and C represent wide-precision planting pattern and conventional-cultivation planting pattern. I1, I2, and I3 represent irrigation (120 mm) at the jointing stage, irrigation (60 mm) at the jointing and heading stages, and irrigation (40 mm) at the jointing, heading, and milking stages. WI1, WI2, and WI3 represent wide-precision planting pattern with irrigation (120 mm) at the jointing stage, irrigation (60 mm) at the jointing and heading stages, and irrigation (40 mm) at the jointing, heading, and milking stages; CI1, CI2, and CI3 represent conventional-cultivation planting pattern with irrigation (120 mm) at the jointing stage, irrigation (60 mm) at the jointing and heading stages, and irrigation (40 mm) at the jointing, heading, and milking stages, respectively.

### Measurements

#### Tillers number

For both growing seasons, tiller number was estimated at the wintering, jointing, and heading stages. At each of these stages, 20 cm sections of two rows were selected in each sub-plot and the plants inside these areas were counted to determine tiller number (plants·m^2^). Tiller extinction rate was defined as follows:

$$Tiller\;extinction\;rate\;(\%)=\frac{A-B}{A}\times 100$$

Tiller extinction rate, where, A (plants·m^-2^) is the tiller number at the jointing stage, and B (plants·m^-2^) is the tiller number at the heading stage.

#### Soil water content

The volumetric soil water content of the planting zone was measured for every 10 cm section of soil, down to 160 cm using a CNC503D neutron moisture meter (Super Energy. Nuclear Technology Ltd., Beijing, China). The water content of the top 20 cm of soil was also measured using the oven-drying method. Soil was oven-dried at 105°C until it became a consistent weight, and then pre-and post-drying weights were compared to determine the water content. This measurement was repeated after all irrigation treatments and major precipitation events. Both methods (volumetric measurements and oven-drying) were used to measure total soil water.

#### Yield components and grain yield

When the winter wheat had reached maturity, two rows (1.5 m each) were selected at random in each experimental plot to measure the spike number, 1000-kernel weight, and grain yield. The plants were harvested manually and air-dried. Additionally, twenty plants were harvested to count the kernel number per spike.

#### Water use efficiency

Water use efficiency (WUE) was defined as follows [16,17]:

$$WUE=\frac{Y}{ET}$$

Where, Y (kg·m^−2^) is the winter wheat grain yield, and ET (mm) is the evapotranspiration during the winter wheat growing seasons. ET was defined as follows [18,19]:

ET=I+P−R−D−ΔS

Where, I (mm) is the irrigation water amount; P (mm) is precipitation, which was measured at the on-site weather station using a standard rain gauge; R (mm) is the surface runoff, which was assumed as non-significant since concrete slabs were placed around each plot; D (mm) is the downward flux below the crop root zone, which was defined as insignificant since soil moisture measurements indicated that drainage at the sites were negligible; and △S (mm) is the change in water storage in the soil profile that is exploited by crop roots (initial soil water content minus soil water content at the end of the growing season).

### Statistical analysis

The effect of the treatments was analyzed using ANOVA (α = 0.05). Multiple comparisons were generated using LSD tests (α = 0.05). Two way ANOVA for interactions of planting pattern × irrigation frequency on grain yield and yield components, soil water consumption, and total water consumption. Prior to the analysis of variance, we tested for normality and homogeneity of variances.

## Results

### Precipitation

In the 2013–2014 and 2014–2015 winter wheat growing seasons, total recorded precipitation was 159 mm and 170mm (Table 2), which was lower than the average seasonal precipitation (the last 50 years) by 85 mm and 74 mm, respectively. In both growing seasons, precipitation occurred mainly from April to May, accounting for 70% and 69% of the total annual precipitation, respectively.

**Table 2:** Precipitation (mm) in the 2013–2014 and 2014–2015 winter wheat growing seasons.

Growing seasons	Oct	Nov	Dec	Jan	Feb	Mar	Apr	May	Jun	Total
2013–2014	4	27	0	0	17	0	33	78	0	159
2014–2015	3	27	2	7	12	2	70	43	4	170

The precipitation in Oct was calculated the sum of precipitation from sowing date to the end of the month. The precipitation in Jun was calculated from 1 Jun to harvested day. The mean annual precipitation in this area in the last 50 years was 697 mm.

### Tiller number

**Fig 2:**
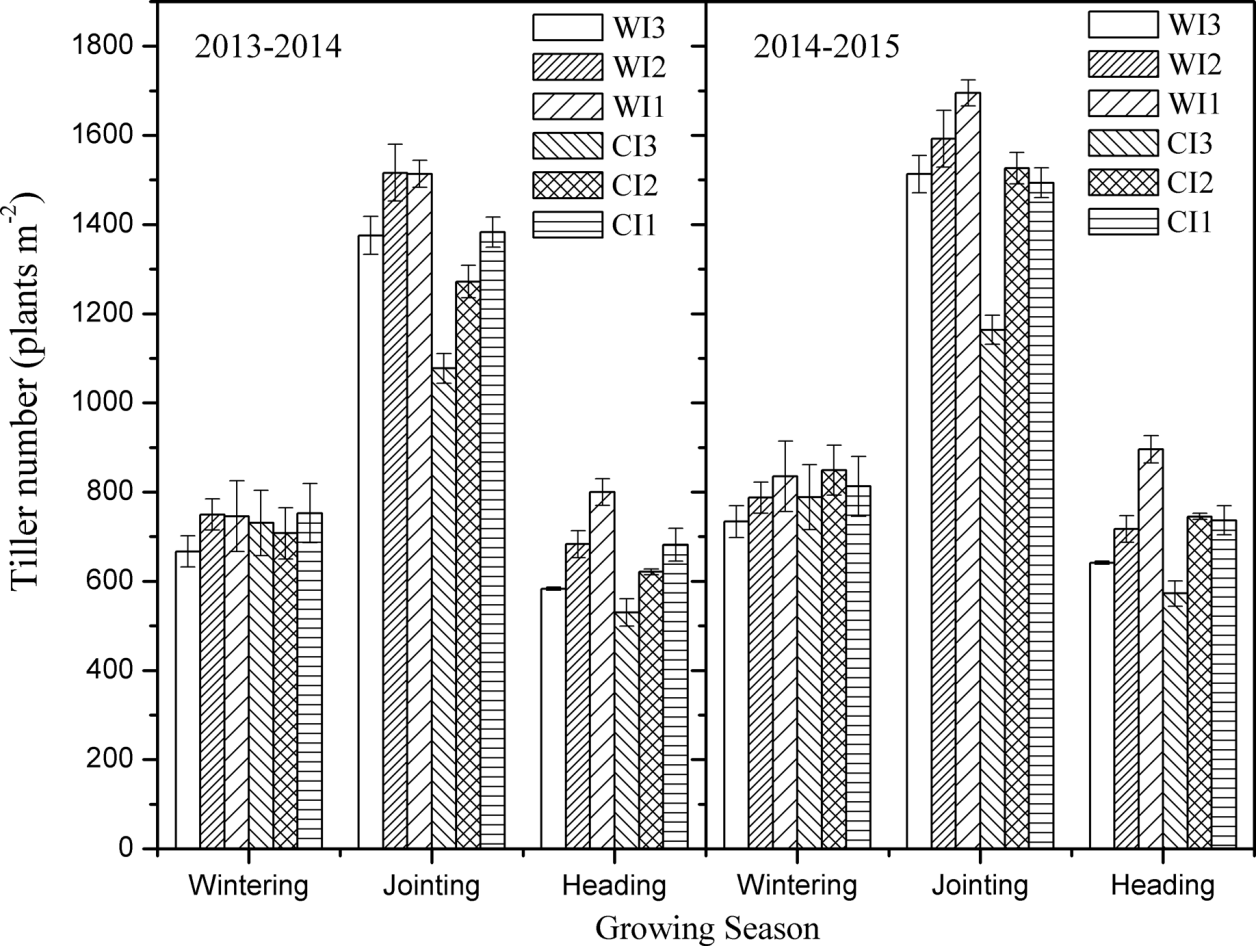
Tiller number in the 2013–2014 and 2014–2015 winter wheat growing seasons. WI1, WI2, and WI3 represent wide-precision planting pattern with irrigation (120 mm) at the jointing stage, irrigation (60 mm) at the jointing and heading stages, and irrigation (40 mm) at the jointing, heading, and milking stages; CI1, CI2, and CI3 represent conventional-cultivation planting pattern with irrigation (120 mm) at the jointing stage, irrigation (60 mm) at the jointing and heading stages, and irrigation (40 mm) at the jointing, heading, and milking stages, respectively. Vertical bars are standard errors.

Both planting pattern and irrigation frequency had significant effects on winter wheat tiller number (Fig 2). At the wintering stage, there were no significant differences among treatments. However, at the jointing stage, the tiller number reached the maximum, and there were significant differences between the different planting patterns (W and C) under the same irrigation regimes. In 2013–2014 and 2014–2015, tiller number was significantly higher (18% and 15%, respectively) in the wide-precision planting pattern than in the conventional-cultivation planting pattern. After the jointing stage, the tiller number declined with time. At the heading stage, reduced irrigation frequency reduced tiller die-off. Compared with I3, the tiller numbers were significantly higher in I1 (33% in 2013–2014 and 35% in 2014–2015) and in I2 (17% in 2013–2014 and 21% in 2014–2015). Due to the different irrigation amounts at the jointing stage, there were significant differences in tiller extinction rate among the treatments. For both planting patterns, I3 resulted in the highest tiller extinction rate, followed by I2, and then I1; therefore, reducing irrigation frequency may have improved the tiller number in both planting patterns. However, in both growing seasons, irrespective of irrigation frequency, tiller number was significantly higher in the wide-precision planting pattern than in the conventional-cultivation planting pattern.

### Yield components and grain yield

Yield components and grain yield were recorded for both growing seasons (Table 3). Irrigation frequency had a significant influence on spike number, it was significantly higher (12% in 2013–2014 and 10% in 2014–2015) in I1 than in I3, and significantly higher (6% in 2013–2014 and 5% in 2014–2015) in W than in C. As for the 1000-kernel weight, with a 10% (2013–2014) and 8% (2014–2015) lower weight in I1 than in I3. Incontrast, there were no significant differences between the two planting patterns for the same measurement. For the total irrigation amount (120 mm), kernel numbers per spike was not significantly affected by either planting pattern or irrigation frequency. Irrigation frequency had a significant influence on the 1000-kernel weight, with a 10% (2013–2014) and 8% (2014–2015) lower weight in I1 than in I3. In contrast, there were no significant differences between the two planting patterns for the same measurement. As for spike number, it was significantly higher (12% in 2013–2014 and 10% in 2014–2015) in I1 than in I3, and significantly higher (6% in 2013–2014 and 5% in 2014–2015) in W and C.

**Table 3:** Yield compositions and grain yield in the 2013–2014 and 2014–2015 winter wheat growing seasons.

Treatments	Spike number(spikes·m^-2^)	Kernel numbers per spike(kernel·spike^-1^)	1000-kernel weight(g)	Grain yield (g·m^-2^)
**2013–2014**				
By planting pattern				
W	467a	36a	45a	716a
C	442b	37a	46a	689b
By irrigation				
I3	429c	37a	46a	693b
I2	455b	35a	48a	721a
I1	479a	38a	43b	693b
Interaction				
WI3	451c	37ab	47ab	694c
WI2	458bc	35ab	46ab	734a
WI1	492a	36ab	43b	719ab
CI3	407d	38ab	46ab	693c
CI2	452bc	35b	49a	707bc
CI1	467b	39a	44b	667d
**2014–2015**				
By planting pattern				
W	487a	40a	50.a	793a
C	465b	39a	50a	723b
By irrigation				
I3	453c	39a	52a	737b
I2	477b	39a	49b	805a
I1	498a	41a	48c	731b
Interaction				
WI3	462cd	42a	53a	758c
WI2	490b	38ab	49bc	828a
WI1	508a	41ab	48c	792b
CI3	444d	36b	51ab	715d
CI2	464c	40ab	49bc	783bc
CI1	488b	41ab	48b	671e

W and C represent wide-precision planting pattern and conventional-cultivation planting pattern. I1, I2, and I3 represent irrigation (120 mm) at the jointing stage, irrigation (60 mm) at the jointing and heading stages, and irrigation (40 mm) at the jointing, heading, and milking stages. WI1, WI2, and WI3 represent wide-precision planting pattern with irrigation (120 mm) at the jointing stage, irrigation (60 mm) at the jointing and heading stages, and irrigation (40 mm) at the jointing, heading, and milking stages. CI1, CI2, and CI3 represent conventional-cultivation planting pattern with irrigation (120 mm) at the jointing stage, irrigation (60 mm) at the jointing and heading stages, and irrigation (40 mm) at the jointing, heading, and milking stages, respectively. In each growing season, values followed by the same letter in the same column, do not differ significantly (LSD, P < 0.05) standard deviation.

Grain yield was significantly affected by both planting pattern and irrigation frequency. Grain yield was significantly higher (4% in 2013–2014 and 10% in 2014–2015) in W than in C. The grain yield in I2 was significantly higher than in I1 (4% in 2013–2014 and 10% in 2014–2015) and in I3 (4% in 2013–2014 and 9% in 2014–2015).

In both growing seasons, significant (LSD, P = 0.05) interactions between planting pattern and irrigation frequency occurred in spike number, kernel numbers per spike, 1000-kernel weight, and grain yield. The results revealed that the wide-precision planting pattern, irrigated (60 mm) at both the jointing and heading stages, resulted in the highest grain yield, and this yield could be mainly attributed to greater tiller survival.

### Water consumption

Soil water consumption and water consumption were measured during the 2013–2014 and 2014–2015 winter wheat growing seasons (Table 4). For both planting patterns, soil water consumption significantly decreased with decreased irrigation frequency. Compared with I3, the soil water consumption in I1 and I2 were significantly lower (23% and 5% in 2013–2014, and 24% and 10% in 2014–2015, respectively). However, under the same irrigation frequencies, there were no significant differences in the soil water consumption between the two planting patterns.

**Table 4:** Water consumption (mm) of winter wheat in the 2013–2014 and 2014–2015 winter wheat growing seasons.

Treatments	Soil water consumption	Water consumption
**2013–2014**		
By planting pattern		
W	84a	362a
C	85a	364a
By irrigation		
I3	93a	372a
I2	89b	367b
I1	71c	350c
Interaction		
WI3	91ab	369ab
WI2	88b	366b
WI1	72c	351c
CI3	96a	374a
CI2	90b	368b
CI1	71c	349c
**2014–2015**		
By planting pattern		
W	81a	370a
C	83a	372a
By irrigation		
I3	72a	381a
I2	84b	372b
I1	70c	359c
Interaction		
WI3	92a	381a
WI2	83b	372b
WI1	68c	357c
CI3	92a	381a
CI2	84b	373b
CI1	72c	361c

W and C represent wide-precision planting pattern and conventional-cultivation planting pattern. I1, I2, and I3 represent irrigation (120 mm) at the jointing stage, irrigation (60 mm) at the jointing and heading stages, and irrigation (40 mm) at the jointing, heading, and milking stages. WI1, WI2, and WI3 represent wide-precision planting pattern with irrigation (120 mm) at the jointing stage, irrigation (60 mm) at the jointing and heading stages, and irrigation (40 mm) at the jointing, heading, and milking stages. CI1, CI2, and CI3 represent conventional-cultivation planting pattern with irrigation (120 mm) at the jointing stage, irrigation (60 mm) at the jointing and heading stages, and irrigation (40 mm) at the jointing, heading, and milking stages, respectively. In each growing season, values followed by the same letter in the same column, do not differ significantly (LSD, P < 0.05) standard deviation. In 2013–2014 and 2014–2015 growing seasons, precipitation was 159 and 170 mm, respectively.

Similar results were found for total water consumption. Compared with I3, the total water consumption in I1 and I2 was significantly lower (6% and 1% in2013–2014, and 6% and 2% in 2014–2015, respectively). There was no significant difference in total water consumption between the two planting patterns.

In both growing seasons, no significant (LSD, P = 0.05) interactions between planting pattern irrigation frequency occurred in soil water consumption and total water consumption. Regardless of the planting pattern, total water consumption of winter wheat significantly decreased with decreased irrigation frequency.

### Water use efficiency

**Fig 3:**
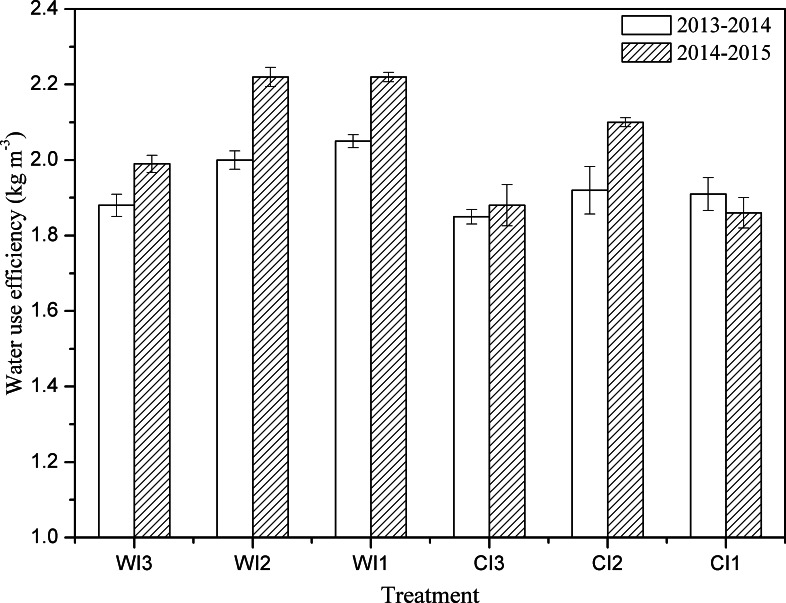
WUE in the 2013–2014 and 2014–2015 winter wheat growing seasons. WI1, WI2, and WI3 represent wide-precision planting pattern with irrigation (120 mm) at the jointing stage, irrigation (60 mm) at the jointing and heading stages, and irrigation (40 mm) at the jointing, heading, and milking stages; CI1, CI2, and CI3 represent conventional-cultivation planting pattern with irrigation (120 mm) at the jointing stage, irrigation (60 mm) at the jointing and heading stages, and irrigation (40 mm) at the jointing, heading, and milking stages. Vertical bars are standard errors.

WUE was calculated for both winter wheat growing seasons (Fig 3). In the first growing season, WUE was significantly higher (5%) in the wide-precision planting pattern than in the conventional-cultivation planting pattern, and significantly higher in I1 (6%) and in I2 (5%) than in I3. Similarly, in the second growing season, WUE was significantly higher in the wide-precision planting pattern (10%), and significantly higher in I1 (6%) and in I2 (12%) than in I3. Assuming a total irrigation amount of 120 mm, a reduced irrigation frequency could significantly increase WUE in wide-precision planting patterns. Considering that there’s no significant difference between WI1 and WI2 for WUE, WI2 could significantly increase spike number, while improving grain yield.

## Discussion

Based on our results, we propose that the wide-precision planting pattern, irrigated (60 mm) at the jointing and heading stages be adopted as the winter wheat production system in semi-humid regions. In wide-precision planting pattern, WUE increased with decreased irrigation frequency, possibly resulting from root development. It is understood that root development directly affects water uptake [20, 21]. For example, at the jointing stage of our study, I3, compared to I1 and I2, was under water deficit conditions, and arid soil conditions are known to promote elongation of the root system of winter wheat. This elongation allows the crop to process soil water more efficiently [22], further develop its root system, and increase its soil water uptake, which makes up for the lack of irrigation water at the jointing stage. This potentially explains why we observed an increase in water consumption with increased irrigation frequency. I1 and I2 were under water deficit conditions at the milking stage. Roots grow slowly and even undergo senescence during this stage, thereby reducing the soil water consumption of winter wheat. Consequently, this may be one reason why WUE increased with decreased irrigation frequency in the wide-precision planting pattern.

In winter wheat, the most important period for tiller survival and for the formation of yield components is after the jointing stage [23]. Under middle-yielding conditions, the spike number is a determining component of grain yield. Compared with I3, both I1 and I2 had a higher irrigation amount at the jointing stage, and this extra water could have enable more winter wheat tillers to survive [24]. The differences in tiller numbers among the treatments would have greatly affected winter wheat yield components. According to [25], compared with the conventional-cultivation planting pattern, the wide-precision planting pattern had a higher spike number per plant. Hence, reduced irrigation frequency, more tillers were survived in wide-precision planting pattern than in conventional-cultivation planting pattern, it’s different from the previous researches [5, 6, 7]. When taking both the planting patterns and irrigation frequencies into account, WI1 and WI2 produced a higher spike number, resulting in an overall higher grain yield than the other treatments.

Winter wheat grain filling relies on two key parts: 1) materials formed before flowering, temporarily stored in vegetative organs in the grain filling stage, and then transported to the seed; and 2) materials formed after flowering. Winter wheat experiencing a water deficit has an effective compensation mechanism within, with photosynthetic products being produced before flowering, and reproductive products (i.e., grain) being produced after flowering [4, 26]. After reducing irrigation frequency, winter wheat was under water deficit conditions during the late stages of the growing season. As it was late in the growing season, plants under drought stress translocated photosynthetic products stored in the vegetative organs to seeds, consequently improving water use efficiency.

Since all the treatments had the same irrigation amount and precipitation, the differences in total water consumption among the different treatments were mainly determined by soil water consumption. Under field conditions, both soil evaporation and leaf transpiration could affect soil water consumption. In the early stages of winter wheat growth, tiller number was much higher in the wide-precision planting pattern than in the conventional-cultivation planting pattern, which means that overall ground coverage was also higher in the wide-precision planting pattern, and this translates to reduced soil evaporation. In the later growth stage, Zhao et al. [12] found that the leaf area index and the leaf transpiration rate were much higher in the wide-precision planting pattern than in the conventional-cultivation planting pattern. Therefore, a positive effect of the wide-precision planting pattern was a decrease in soil evaporation and an increase in leaf transpiration. Although there were no significant differences in total water consumption between the two planting patterns, ineffective water consumption (i.e., soil evaporation) was converted to effective water consumption (i.e., leaf transpiration) in the wide-precision planting pattern. This maybe another reason why the wide-precision planting pattern had the greater WUE.

Irrigation frequency and planting pattern greatly influence grain yield and WUE. This is principally because of the impact of irrigation frequency on tiller extinction, which in turn influences spike numbers. Lang et al. [27] studied the effect of delayed irrigation on the WUE of winter wheat planted in a wide-precision planting pattern. The study revealed that irrigation (60 mm) 10 days after the jointing stage achieved a reasonable WUE and grain yield, which was predominantly due to an increase in spike number. We could assume that delaying the full irrigation quantity (120 mm) 10 days after jointing stage would further decrease tiller extinction; however, this topic requires further exploration.

In both growing seasons, precipitation was below the seasonal average. In drier seasons like these, reducing irrigation frequency, while still using the total amount of irrigation (120 mm), could increase winter wheat grain yield and WUE. In years of average precipitation, it is not clear if reducing irrigation frequency would have the same impact on grain yield and WUE. However, at our study area, annual precipitation has been trending downwards over the last 40 years [28], and therefore dry years are becoming more common. Our study is therefore relevant in ensuring stable, high-yielding winter wheat production into the future. On the other hand, only one wheat cultivars was tested in our study and there are many different cultivars developed for dry regions [29]. For this reason, different winter wheat cultivars may show different responses to the different planting patterns and irrigation frequencies. This topic requires further exploration. We should also investigate other issues, such as water leakage, adaptability of deep sandy soils, and reasonable shoot growth under different irrigation conditions.

## Conclusion

A combination of decreased irrigation frequency and wide-precision planting pattern significantly increased winter wheat WUE, and this was due to reduced soil water consumption. Tiller number, and in turn, grain yield, were significantly higher in the wide-precision planting pattern than in the conventional-cultivation planting pattern. In the semi-humid regions, a wide-precision planting pattern, irrigated60 mm at the jointing and 60 mm at the heading stage should promote water use efficiency and higher-yields of winter wheat.

## Supporting Information

S1 Dataset S1 Dataset for precipitation. (XLSX)

S2 Dataset S2 Dataset for tiller number. (XLSX)

S3 Dataset S3 Dataset for water consumption. (XLSX)

S4 Dataset S4 Dataset for WUE. (XLSX)

S5 Dataset S5 Dataset for yield compositions and grain yield. (XLSX)
